# Type 2 diabetes is not associated with excess risk of periprosthetic joint infection in obese patients undergoing total hip arthroplasty

**DOI:** 10.1186/s12891-026-09568-5

**Published:** 2026-02-03

**Authors:** Aurora Tasa, Johanna Vinblad, Jonatan Nåtman, Jonatan Tillander, Fredrik Nyberg, Martin Englund, Ola Rolfson

**Affiliations:** 1 Department of Orthopedics, Institute of Clinical Sciences, Sahlgrenska Academy at the University of Gothenburg, Gothenburg, Sweden; 2 Centre of Registers Västra Götaland, Gothenburg, Sweden; 3 Sahlgrenska University Hospital, Gothenburg, Sweden; 4 Department of Infectious Diseases, Institute of Biomedicine, Sahlgrenska Academy at the University of Gothenburg, Gothenburg, Sweden; 5 School of Public Health and Community Medicine, Institute of Medicine, Sahlgrenska Academy at the University of Gothenburg, Gothenburg, Sweden; 6 Department of Orthopedics, Institute of Clinical Sciences, Faculty of Medicine, Lund University, Lund, Sweden

**Keywords:** Osteoarthritis, Total hip arthroplasty, Diabetes, Obesity

## Abstract

**Background:**

Obesity and type 2 diabetes (T2DM) are considered risk factors for complications following total hip arthroplasty (THA). How these risk factors separately or in combination influence the risk of reoperation due to periprosthetic joint infection (PJI) or all-cause reoperation is not fully known. We sought to investigate the 2-year risk of reoperation following THA due to osteoarthritis (OA) among people with and without the metabolic risk factors, T2DM and obesity.

**Methods:**

We linked all patients with THA registered during 2008–2019 in the Swedish Hip Arthroplasty Register (SHAR) to the National Diabetes Register. The risk of reoperation was analysed by applying Kaplan–Meier (KM) and multivariable Cox regression analyses. Subgroup analyses were performed to explore the effect modification by obesity. After THA exclusions (such as bilateral THA observations and other diagnoses than OA) there were 14,512 individuals identified with T2DM and 116,579 without. The primary outcome was reoperation due to PJI within two years after THA. The secondary outcome was reoperation due to any cause within two years after THA.

**Results:**

2-year 1–KM reoperation estimate for reoperation due to PJI was 1.69% with 95% confidence interval (95%CI) 1.47–1.90 among individuals with T2DM and 1.02% (95%CI 0.96–1.08) among those without T2DM. The 1–KM reoperation estimate for reoperation due to any cause was 2.67% (95%CI 2.40–2.94) for the T2DM group, and 1.99% (95%CI 1.91–2.07) for non-T2DM. However, adjusted for Body Mass Index (BMI), T2DM was not a statistically significant risk factor (infection-related reoperation: adjusted hazard ratio (aHR) 1.1 with 95% confidence interval (95%CI) 0.9–1.3; all-cause reoperation: aHR 1.0, 95%CI 0.9–1.2). In the BMI subgroup analyses, T2DM associated increased risk of reoperation due to PJI was limited to normal/underweight individuals (aHR 1.6 95%CI 1.1–2.4). In the subgroups with overweight or obesity, T2DM was neither associated with increased risk of all-cause reoperation nor reoperation due to PJI.

**Conclusions:**

Overall, T2DM is not an independent risk factor for reoperation when adjusted for BMI. However, T2DM constitutes a minor risk factor in individuals of normal/underweight. Risk of reoperation was more strongly associated with BMI rather than T2DM, suggesting that the additional risk observed in obese patients is likely influenced by multiple interrelated factors beyond T2DM alone.

**Supplementary Information:**

The online version contains supplementary material available at 10.1186/s12891-026-09568-5.

## Introduction

Osteoarthritis (OA) is the most common joint disorder and form of arthritis. Hip OA is among the most prevalent disabling conditions worldwide, and an increasing number of people need to undergo hip replacement surgery due to insufficient effect of non-surgical treatments and increasing life expectancy [1]. Sweden has one of the highest rates of total hip arthroplasty (THA), with around 20,000 operations performed annually. The most common indication for primary THA is OA, and the proportion of primary OA as indication has increased from 75% in 2000 to 82% in 2019 [2]. It is estimated that a quarter of the Swedish population aged over 45 years suffers from pain and physical activity restrictions linked to OA [3]. Possible links between manifestation of OA and type 2 diabetes mellitus (T2DM) are physical inactivity, obesity, and hyperglycemic toxicity [4]. According to data from the Swedish National Diabetes Register (NDR), approximately 5.5% of the population has diabetes, and T2DM makes up a majority of 90% [5]. The prevalence of T2DM is projected to continue as a result of changes in age structure, population size and obesity, together with decreased mortality among people with diabetes [6]. Obesity has been suggested to play an important role by causing stress on joints due to excess mechanical overload but also by causing low-grade inflammation that originates from adipose tissue [7, 8]. Together, obesity, diabetes and OA may create a vicious circle where the factors build on and reinforce each other.

### Rationale

Currently, primary THA is considered a routine procedure and is performed more frequently in all age groups according to data from the Swedish Hip Arthroplasty Register (SHAR) annual report 2019 [2]. A majority of patients do well after THA, but some suffer postoperative complications that require further surgical interventions. A register-based observational study showed that an increasing body mass index (BMI) was associated with increased risk of reoperation and revision following THA [9]. BMI is strongly associated with increased rates of deep PJI, but also with elevated risk for hip dislocations and wound infections [10]. Previous studies have also shown that diabetes contributes to a higher risk of perioperative adverse events. Kebaish et al. demonstrated that especially insulin-dependent diabetes mellitus resulted in elevated risk for adverse events, particularly at BMI greater than 40 kg/m2 [11]. Webb et al. reported the same connection between insulin-dependent diabetes and elevated risk for adverse events and emphasized the importance of acknowledging insulin-dependency as an independent risk factor for complications following THA [12]. It is hypothesized that higher BMI alongside a thicker adipose tissue layer complicates both surgery and healing, contributing to the risk of developing postoperative complications, mainly infections. Based on this, there have been discussions whether more stringent BMI restrictions should be employed in the decision-making before THA, particularly for people with comorbidities. However, there is insufficient knowledge on the risks of obesity and its relationship with other comorbidities in THA surgery, and further research is needed [13]. Our aim was to investigate if the combination of both having diabetes and being obese increases the risk even more.

### Study questions

1. What is the risk of reoperation due to all causes and specifically due to PJI within two years following THA for individuals with T2DM compared to individuals without type 2 diabetes (non-T2DM)?
2. How does T2DM affect the risk of reoperation due to PJI stratified on BMI?

## Patients and methods

### Study design and setting

The Swedish Osteoarthritis and Diabetes (SOAD) cohort is based on prospectively obtained individual-level data from three nationwide registers: the Swedish Arthroplasty Register (SAR), the National Swedish Diabetes Register (NDR) and the Swedish Osteoarthritis Register (SOAR). This study was conducted within the SOAD project with a focus on SAR and NDR. SAR is a national quality register collecting data on hip replacement operations in Sweden since 1979. In SAR, data on patient characteristics, operative features and all causes of reoperations after THA are collected. Reoperation rate within two years is used as a quality indicator for primary THA. The most common reasons for early reoperation are infection and dislocation, and interestingly, an increasing proportion of THA is reoperated due to infections [2]. Over the last decade, the completeness for primary THAs has stayed between 97% and 99%. The corresponding completeness for revisions was 91% in Sweden in 2018 [2]. NDR was launched in 1996 and includes patient-level data on clinical characteristics, laboratory analyses, risk factors, complications, and medications of individuals with diabetes. The completeness rate for NDR was 88% in 2019 [5].

### Participants/study subjects

**Fig. 1:**
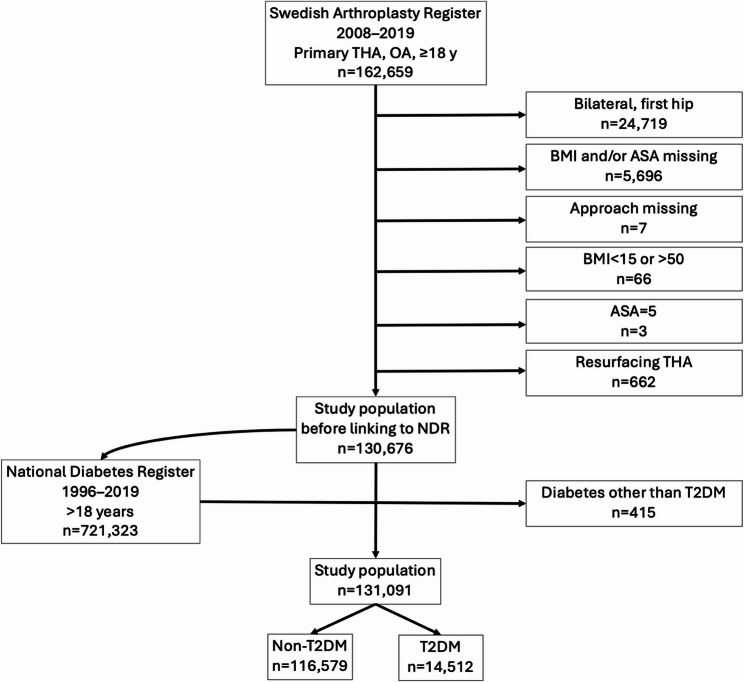
Flowchart of study population selection who underwent THA for primary osteoarthritis in Sweden 2008–2019. THA (total hip arthroplasty). BMI (Body Mass Index). ASA (American Society of Anesthesiology classification system). T2DM (type 2 diabetes mellitus)

There were 162,659 elective THAs identified from SHAR between 2008 and 2019. To reduce heterogeneity, we applied additional selection criteria to define the study cohort (Fig. 1). All selected individuals were over 18 years of age and the indication for operation was primary OA. Moreover, if a person had undergone bilateral hip replacement, only the later operation was included in the study [14]. In addition, people with any missing measurements, such as for BMI, ASA and surgical approach, were excluded. Due to a low number of observations and potential data errors, we excluded individuals with registered BMI under 15 or over 50, American Society of Anesthesiologists Classification system (ASA) class 5, and all operations with resurfacing prostheses. The selected patients were linked with the data from NDR (Fig. 1).

### Variables, outcome measures, data sources, and bias

The main outcome of interest was reoperation due to PJI within two years after the primary THA (index arthroplasty), defined as an open surgical procedure related to the existing prosthesis. In the dataset, the possible causes leading to reoperation were PJI, aseptic loosening, dislocation, periprosthetic fracture and other reasons as reported by the surgeon at the time of surgery. PJIs were analysed separately. Age, sex, type of fixation, surgical approach, ASA and BMI considered as confounders in the analyses, as previous studies have shown that these factors are associated with both the surgical procedure itself (exposure) and the risk of reoperation outcome [15–17]. The prostheses were categorized into three different groups according to the type of fixation: cemented, uncemented and hybrid prostheses. Surgical approaches were classified into lateral, posterior and other. ASA classes were combined into two groups, ASA 1–2 and ASA 3–4, acknowledging that comorbidities such as diabetes are included in the ASA classification system. We utilized the World Health Organization classification for BMI: <18.5 underweight, 18.5–<25 normal weight, 25–<30 overweight, 30–<35 class 1 obesity and > 35 class 2–3 obesity. Since there were few individuals who were underweight, they were combined with the group of people with normal weight. Overall, if a variable of interest was present in the data more than once, we used the measurement closest to the time point of THA.

### Statistical analysis, study size

Baseline characteristics were reported as mean, median, standard deviation and interquartile range. Categorical variables were summarized as frequencies and percentages. Kaplan–Meier estimates (1-KM) were used to describe the cumulative reoperation rates for the study groups (T2DM vs. non-T2DM). Firstly, KM estimates [with 95% confidence intervals (CI)] for reoperation due to PJI and any cause were computed, respectively. Secondly, we performed subgroup KM analyses of the diabetes effect stratified by BMI. A Cox proportional hazards model was used to analyze the two-year time-to-event risk of reoperation following primary THA. The assumption of proportional hazards was assessed graphically. Adjusted models were adjusted for age, sex, fixation method, surgical approach, ASA and BMI class. In the stratified analyses for reoperation due to PJI within two years following primary THA, people were categorized by their BMI classification. SPSS version 26 and R version 4.0.2 were used for these statistical analyses.

## Results

### Description of study population

After exclusions, 116,579 individuals without type 2 diabetes mellitus (non-T2DM) and 14,512 with T2DM undergoing primary THAs between 2008 and 2019 remained for the analyses (Table 1). The T2DM group was slightly older and included fewer women. As expected, higher ASA classes were more common in the T2DM group. The posterior approach was the most frequently used surgical technique. Cemented fixation was more common among individuals with T2DM. Median BMI was higher in the T2DM group, with fewer individuals in the normal weight category compared to those without T2DM.

**Table 1:**
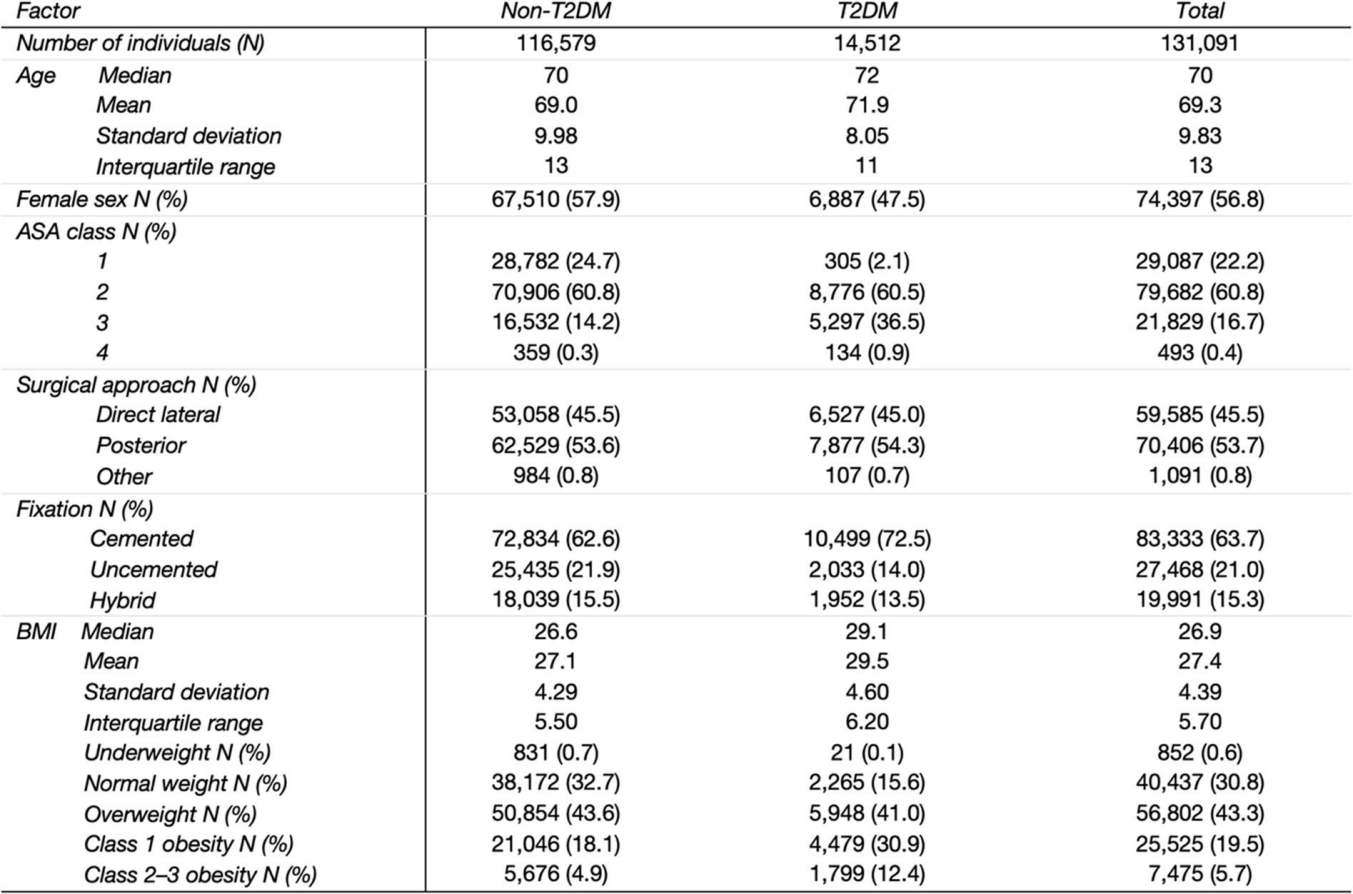
Characteristics of the included patients

Characteristics of patients ≥18 years old who underwent THA for primary osteoarthritis in Sweden 2008–2019, overall and by type 2 diabetes, BMI Body Mass Index classes (kg/m2): <18.5 underweight, 18.5–<25 normal weight, 25–<30 overweight, 30–<35 class 1 obesity, >35 class 2–3 obesity

*THA* Total hip arthroplasty, *OA *Osteoarthritis, *ASA* American Society of Anesthesiology classification system, *Non-T2DM *Non-type 2 diabetes mellitus, *T2DM* Type 2 diabetes mellitus

### Risk of reoperation due to infection and all causes

**Fig. 2:**
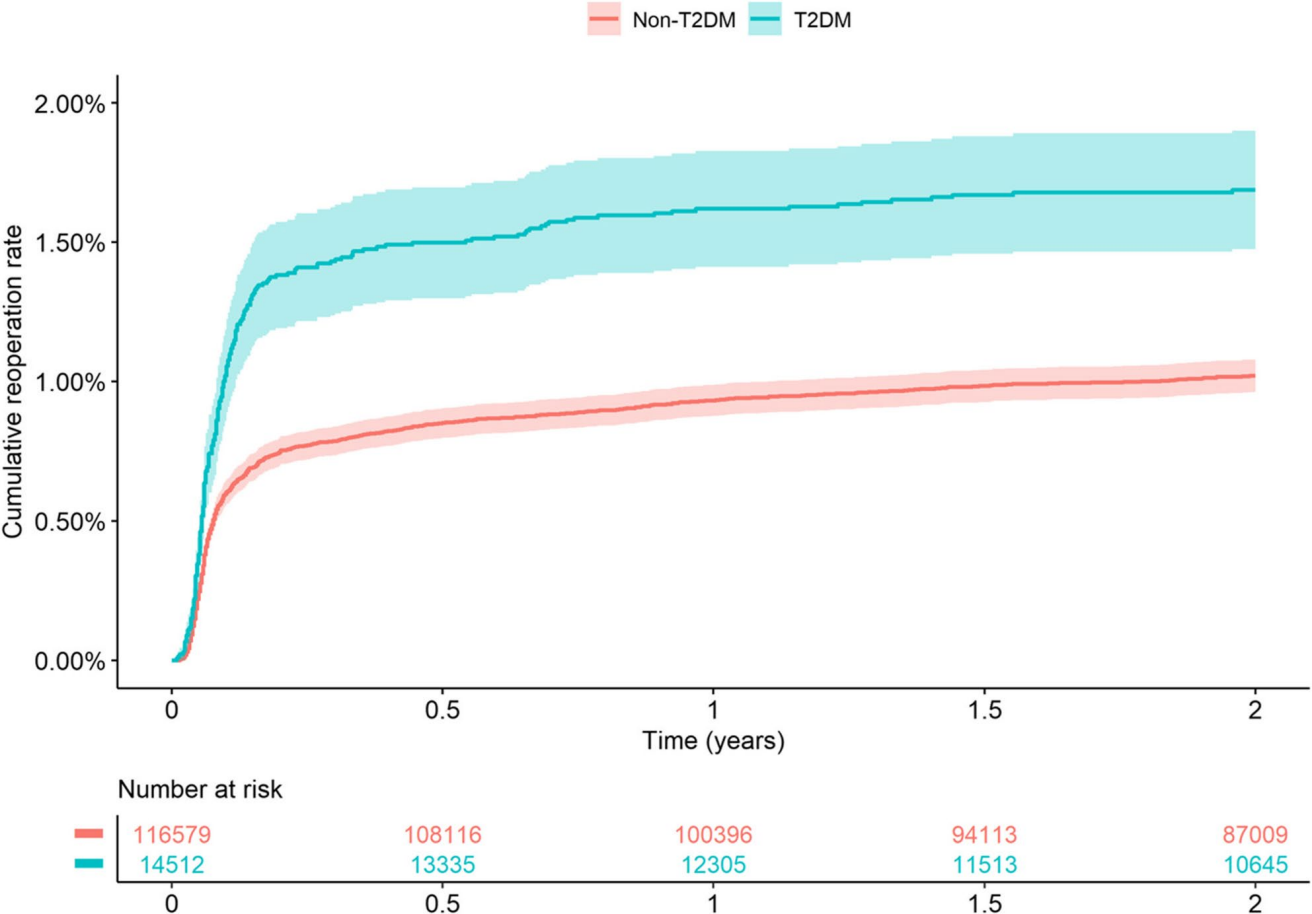
Kaplan–Meier [1-KM, (%)] 2-year cumulative estimates for reoperation due to infection for patients with and without type 2 diabetes who underwent total hip arthroplasty due to osteoarthritis in Sweden 2008–2019. T2DM (type 2 diabetes mellitus). KM (Kaplan–Meier)

**Fig. 3:**
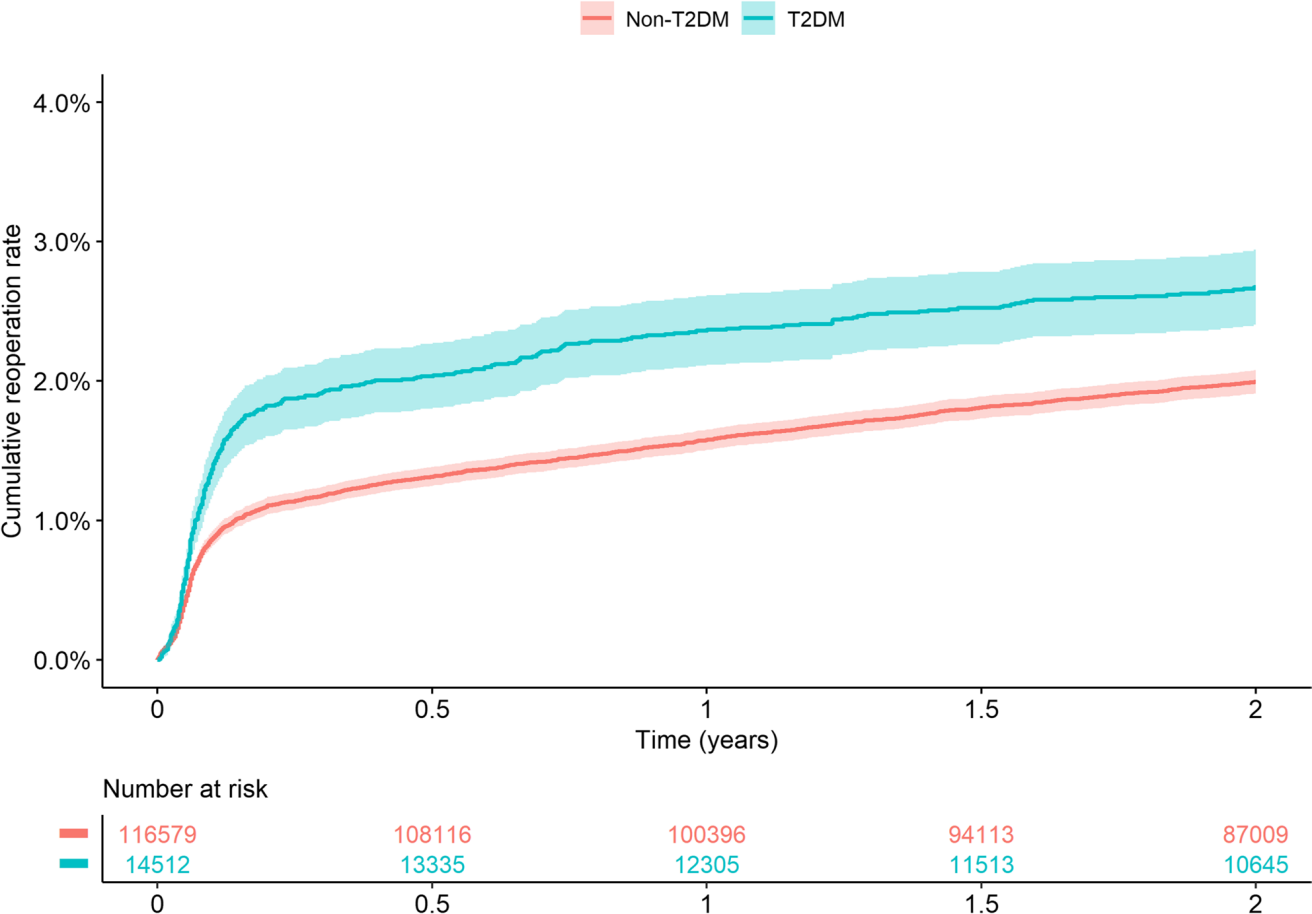
Kaplan–Meier [1-KM (%)] 2-year cumulative estimates for reoperation due to any cause for patients with and without type 2 diabetes who underwent total hip arthroplasty due to osteoarthritis in Sweden 2008–2019. T2DM (type 2 diabetes mellitus). KM (Kaplan–Meier)

**Fig. 4:**
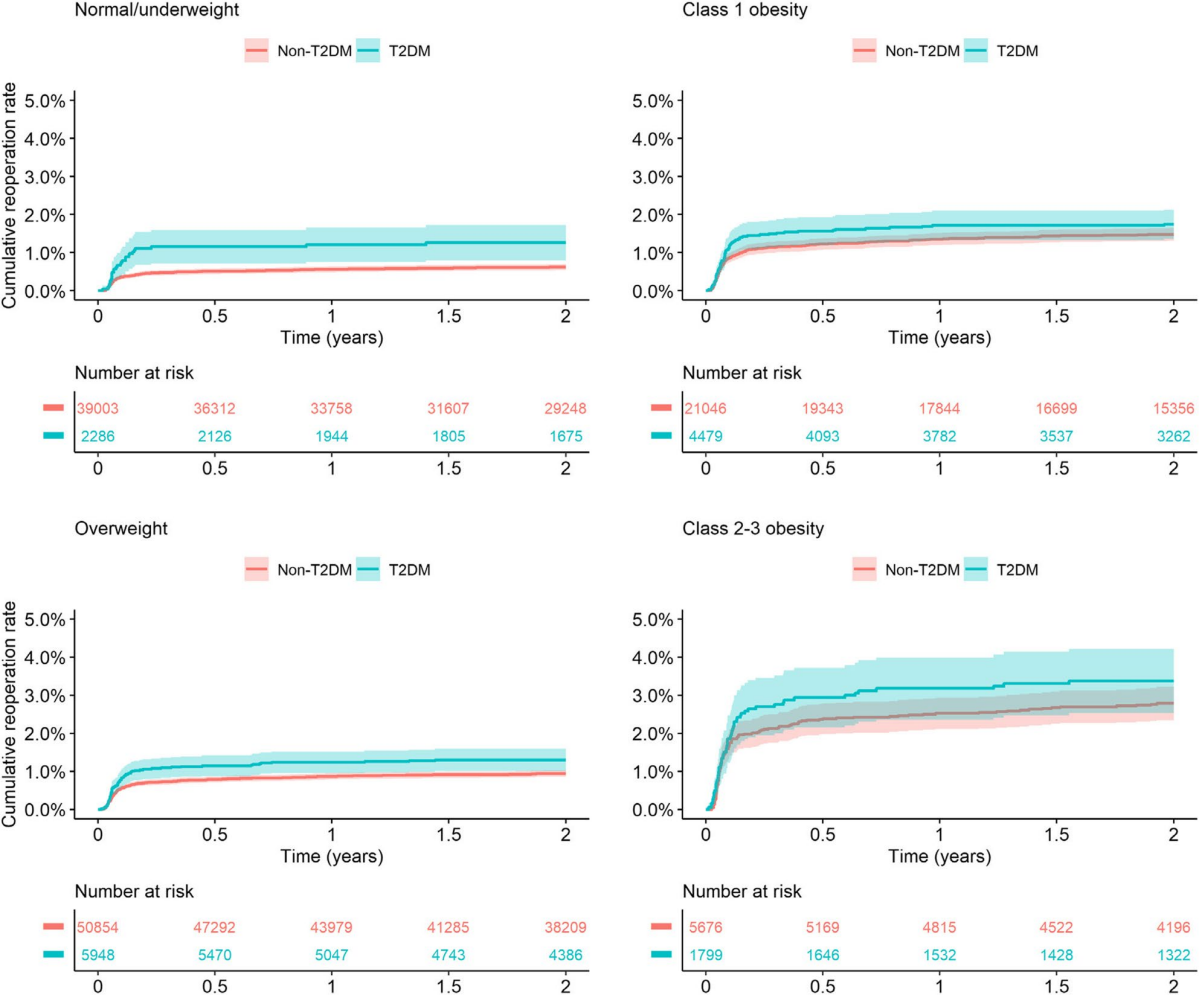
Subgroup Kaplan–Meier [1-KM (%)] 2-year cumulative estimates for reoperation due to infection for patients with type 2 diabetes who underwent total hip arthroplasty due to osteoarthritis in Sweden 2008–2019, stratified by BMI. BMI (Body Mass Index) classes (kg/m2): <18.5 underweight, 18.5–<25 normal weight, 25–<30 overweight, 30–<35 class 1 obesity, >35 class 2–3 obesity. T2DM (type 2 diabetes mellitus). KM (Kaplan–Meier)

The risk of reoperation was elevated in individuals with diabetes. The 2-year cumulative incidence estimate for reoperation due to PJI was 1.69% (95%CI 1.47–1.90) for the T2DM group, whereas in the non-T2DM group it was 1.02% (95%CI 0.96–1.08) (Fig. 2). The cumulative incidence estimates for reoperation within 2 years due to any cause (including PJI) was 2.67% (95%CI 2.40–2.94) for the T2DM group, and 1.99% (95%CI 1.91–2.07) for the non-T2DM group (Fig. 3). In the analyses stratified by BMI, T2DM was associated with a higher risk for reoperation in normal and underweight individuals (Fig. 4). The adjusted Cox regression showed no 2-year risk increase for reoperation due to PJI in T2DM compared to non-T2DM (unadjusted HR 1.68, 95%CI 1.46–1.93; adjusted HR 1.10, 95%CI 0.95–1.27) (Table 2). In addition, diabetes was not a statistically significant risk factor for reoperation due to any cause (adjusted HR 1.03, 95%CI 0.92–1.16) (Table 2).

**Table 2:** Risk of reoperation due to infection and all causes

	HR	95%CI	*p*-value
Reoperation due to infection within 2 years*N* events: 1384			
Unadjusted	1.68	1.46–1.93	< 0.001
Adjusted for age, sex, surgical approach, fixation method, ASA and BMI class	1.10	0.95–1.27	0.197
Reoperation due to all causes within 2 yearsN events: 2557			
Unadjusted	1.37	1.23–1.53	< 0.001
Adjusted for age, sex, surgical approach, fixation method, ASA and BMI class	1.03	0.92–1.16	0.588

Hazard ratios for reoperation due to infection and all causes within two years for patients with type 2 diabetes who underwent total hip arthroplasty due to osteoarthritis in Sweden 2008–2019

*T2DM *Type 2 diabetes mellitus*, ASA* American Society of Anesthesiologists classification system, *BMI* Body Mass Index, *HR* Hazard ratio, *CI* Confidence interval

### Risk of reoperation due to infection stratified by BMI

The adjusted HR was elevated for individuals with diabetes who were normal/underweight (HR 1.62; 95%CI 1.08–2.41) (Table 3). Risks were not appreciably increased: in overweight (HR 1.10; 95%CI 0.86–1.42), class 1 obesity (HR 1.00; 95%CI 0.77–1.30) and class 2–3 obesity (HR 1.09; 95%CI 0.80–1.48) (Table 3).

**Table 3:** Risk of reoperation due to infection stratified by BMI

Reoperation due to infection	Normal/underweight	Overweight	Class 1 obesity	Class 2–3 obesity
N individuals	41,289	56,802	25,525	7475
N events	260	538	375	211
	HR [95%CI]	HR [95%CI]	HR [95%CI]	HR [95%CI]
Non-T2DM	1.00	1.00	1.00	1.00
T2DM	1.62 [1.08–2.41]	1.10 [0.86–1.42]	1.00 [0.77–1.30]	1.09 [0.80–1.48]
Male sex	1.00	1.00	1.00	1.00
Female sex	0.48 [0.35–0.57]	0.47 [0.40–0.57]	0.52 [0.42–0.65]	0.61 [0.46–0.81]
ASA 1–2	1.00	1.00	1.00	1.00
ASA 3–4	1.31 [0.96–1.79]	1.52 [1.23–1.87]	1.18 [0.92–1.51]	1.24 [0.93–1.64]
Lateral approach	1.00	1.00	1.00	1.00
Posterior approach	0.86 [0.67–1.09]	0.69 [0.58–0.82]	0.72 [0.58–0.88]	0.84 [0.64–1.11]
Other approaches	-	0.52 [0.17–1.62]	0.60 [0.15–2.42]	1.95 [0.62–6.18]
Cemented prosthesis	1.00	1.00	1.00	1.00
Uncemented prosthesis	1.16 [0.78–1.72]	1.32 [1.03–1.69]	1.21 [0.91–1.61]	1.16 [0.80–1.68]
Hybrid prosthesis	0.80 [0.53–1.22]	0.83 [0.62–1.10]	0.91 [0.66–1.26]	1.00 [0.66–1.51]
Age	1.03 [1.01–1.04]	1.03 [1.02–1.04]	1.02 [1.01–1.04]	1.02 [1.00-1.03]

Adjusted hazard ratios for reoperation due to infection within two years for patients with type 2 diabetes who underwent total hip arthroplasty due to osteoarthritis in Sweden 2008–2019, stratified by BMI. Models were adjusted for age, sex, fixation method, surgical approach, ASA and BMI class. The reference categories are written in *italics*. BMI (Body Mass Index) classes (kg/m^**2**^): <18.5 underweight, 18.5–<25 normal weight, 25–<30 overweight, 30–<35 class 1 obesity, > 35 class 2–3 obesity

*ASA* American Society of Anesthesiologists classification system, *T2DM* Type 2 diabetes mellitus, *HR* Hazard ratio, *CI* Confidence interval

## Discussion

Our aim was to examine if the combination of T2DM and obesity increases the risk of reoperation due to PJI and any cause following THA. Prevalences of diabetes and obesity are increasing world-wide and their pathophysiological role in OA is non-negligible [18]. Our multivariate analysis shows an additive risk for PJI in underweight and normal weight individuals with T2DM, but not in higher weight classes. Our interpretation is that when it comes to the risk of complications, obesity is a more determinative factor and may overshadow the risks that diabetes would normally incur. To our knowledge, this is a novel finding regarding the relationship between obesity, diabetes, and the risk of PJI.

Diabetes is associated with elevated prevalence of OA, partly due to obesity, but metabolic factors may also play a role. Metabolic OA is considered a subtype of OA linked to T2DM [19]. In a systematic literature review and meta-analysis by Louati et al., the connection between OA and diabetes was demonstrated without convincing causality, likely due to confounding factors, such as obesity [20], which seems to be the most important component in the metabolic syndrome [21]. However, it is also suggested that T2DM is associated with both radiographic and symptomatic OA even when controlling for BMI, indicating that mechanisms beyond obesity may contribute to OA development in patients with T2DM [18]. Consistent with Kebaish et al. [11], we could demonstrate that diabetes resulted in a higher risk of developing postoperative complications compared to non-diabetic individuals. Furthermore, we found that the cumulative incidence of reoperation was higher for those with obesity supporting the findings of Sayed-Noor et al. [9]. A retrospective case-control study by Jahng et al. suggested the potential cumulative risk in concurrent obesity and diabetes and reported that wound complications and reoperations were more likely for people with both diabetes and obesity. However, some results lacked statistical significance, possibly due to small sample sizes [22].

Obesity is generally seen as a strong complicating factor for several medical conditions and treatments. THA is often surgically more demanding on obese patients. Performing THA on obese patients has been associated with longer operation times and therefore higher rates of complications [23, 24]. Nonetheless, there is still some unclarity. Shaparin et al. reported that a higher BMI seemed to predict early postoperative complications in THA, in a non-linear manner, as certain weights seemed to protect from some undesired postoperative outcomes, such as acute blood loss, wound infection or pulmonary embolism [25]. Shaparin et al. refers to ‘’the obesity paradox’’ and emphasizes that further studies are needed in the field of THA and its complications that are related to BMI or other comorbid conditions. Furthermore, some studies have found only minor differences in the complication rates across the BMI classes, which also supports the idea of a non-linear risk [26]. Further research should include other diabetes-related factors and focus on OA phenotypes. More research is needed to better understand the variation in complication rates across BMI categories and their underlying factors.

### Strengths and limitations

This study used data from Swedish nationwide registers with high coverage and data completeness that are widely used in medical research. Register-based research enables large study groups which is a strength of this study. Nonetheless, some underreporting of reoperations to SHAR does exist. The completeness regarding revisions was around 91% in 2018, while for other types of reoperations (constituting some 10% of all reoperations) the corresponding completeness is 70 to 80% [27]. Importantly, systematic bias of under-reporting based on diabetes status is unlikely. The missing data for BMI and ASA cannot be assumed to be missing at random. However, the primary explanation for the missingness pertains to delay in starting registration of these variables when they were introduced in the register in 2008. When we categorized the study groups into several different weight and ASA classes, our sample sizes became lower, which can explain why some results were not statistically significant. It is also important to take into consideration that BMI as an estimate of obesity does not distinguish weight that is associated with adipose tissue from weight that is associated with muscle mass. However, BMI is a convenient and generally accepted measure of obesity in medical research. We included individuals with a recorded T2DM diagnosis prior to their THA operation. However, since NDR only reports the year of T2DM onset, we could not confirm whether the diagnosis was established before the surgery. On the other hand, very few individuals were affected by this possible limitation. In addition, developing diabetes is usually a long process, so we can assume that the condition was already ongoing at the time of the operation. There might have been unknown statistical interactions between the variables that we could not interpret since diabetes itself leads to a higher ASA classification. In addition, other confounding factors may have affected our findings, such as inadequate diabetes management, disease severity, diabetes neuropathy, hypertension, dyslipidemia, smoking and physical inactivity [28]. However, there are guidelines on diabetes management preceding THA that can be assumed to have been followed. Furthermore, it is important to consider that patients undergoing THA have already been assessed by a physician to determine surgical safety, which may lead to selection bias, as individuals with significant obesity or poorly controlled diabetes may be excluded at an early stage. In today’s preoperative assessment prior to THA, glycosylated hemoglobin (HbA1c) is used for assessing glycemic control before surgery as most clinics do not accept patients for surgery until diabetes is well controlled. Therefore, it should be noted that these results consider a selection of reasonably well-regulated diabetic individuals. We had access to clinical measurements, such as HbA1c, but too few recorded measurements in conjunction with the operation made analysis impossible. However, previous studies have shown that HbA1c as a predictive risk factor for postoperative infections following joint replacement surgery is not reliable, and it is hypothesized that there is a connection with the variability of OA phenotypes [19]. Lastly, there is some controversy in the association of hypertension and dyslipidemia with OA [20]. Nevertheless, other diabetes-related risk factors and comorbidities are yet to be explored, and even larger study groups could be an advantage.

## Conclusions

T2DM is not associated with reoperation due to PJI when adjusted for BMI. However, our subgroup analysis suggested that diabetes constitutes a minor risk factor for people with normal or subnormal BMI, whilst in the higher BMI classes, diabetes itself does not seem to affect risk appreciably. Thus, the risk of reoperation due to PJI does not appear to be driven by diabetes but seems to be more associated with BMI. These findings offer important and novel information that affects preoperative risk stratification on both non-diabetic and diabetic individuals. 

## Supplementary Information

Supplementary Material 1.

## Data Availability

The data that support the findings of this study are available from Region Västra Götaland but restrictions apply to the availability of these data, which were used under license for the current study, and so are not publicly available. Data are however available from the authors upon reasonable request and with permission of the Swedish Ethical Review Authority and Region Västra Götaland.
