# Supplementary material for: Type 2 diabetes is not associated with excess risk of periprosthetic joint infection in obese patients undergoing total hip arthroplasty
Source: BMC Musculoskelet Disord. 2026 Feb 3;27:170. doi: 10.1186/s12891-026-09568-5 (PMC12930599; doi:10.1186/s12891-026-09568-5)
Supplement: Supplementary file 1 — Supplementary Material 1. [file 12891_2026_9568_MOESM1_ESM.docx]

Supplements
